# Supplementary material for: The Role of Baseline Total Kidney Volume Growth Rate in Predicting Tolvaptan Efficacy for ADPKD Patients: A Feasibility Study
Source: J Clin Med. 2025 Feb 21;14(5):1449. doi: 10.3390/jcm14051449 (PMC11899928; doi:10.3390/jcm14051449)
Supplement: Supplementary file 1 [file jcm-14-01449-s001.zip › jcm-3399630-supplementary.pdf]

Supplementary Materials:

Supplemental Figure S1. Kidney Volume Trajectory Plots for 32 Subjects. Height adjusted total kidney volume (htTKV) versus age including tolvaptan initiation (grey), TKV growth rates pre- (black) and during tolvaptan (blue). A. Responders had a decrease in TKV growth rate since starting tolvaptan greater than 2% per year. B. Non-responders had an increase in TKV growth rate or a decrease in TKV growth rate less than 1% per year.

A

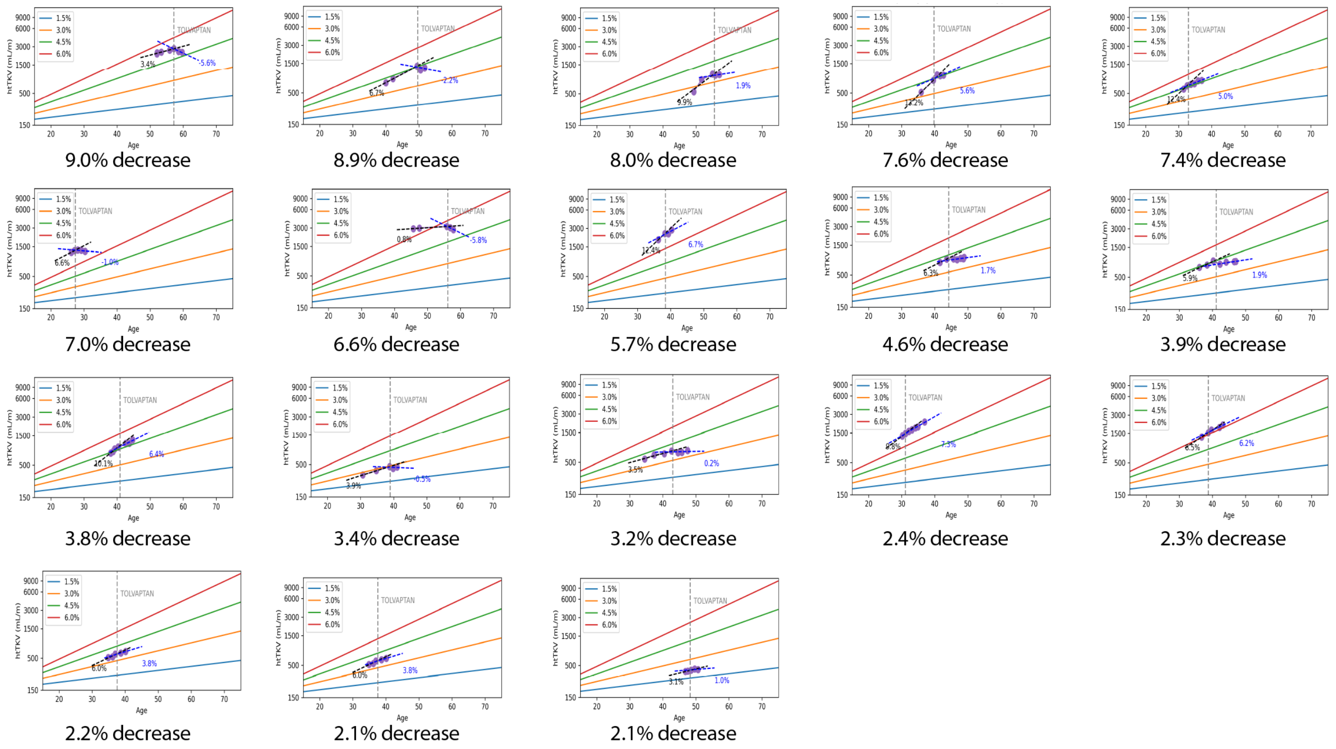

B

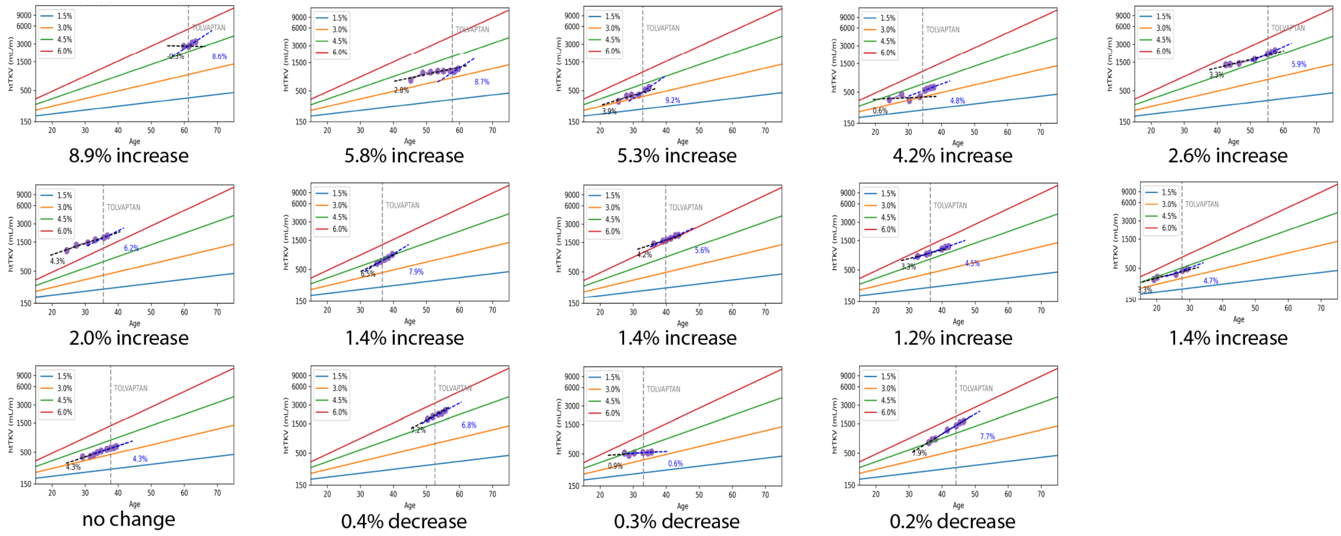

Supplemental Table S1. Comparison of study cohort (*n* = 32) to all patients on tolvaptan.

	**Study Subjects (*n* = 32)**	**Subset of Non-study subjects on tolvaptan (*n* = 32)**	***p*-value**
Age (years)	42 ± 9	41 ± 10	0.87
Sex (male:female, %male)	17:15	16:16, 50%	0.80
Height (m)	1.7 ± 0.1	1.7 ± 0.1	0.46
Weight (kg)	75 ± 16	82 ± 23	0.32
BMI[1] (kg/m^2)	26 ± 4	27 ± 6	0.60
TKV (mL)	1622 [1067, 2715]	1747 [1311, 2544]	0.45
htTKV (mL/m)	975 [589, 1578]	963 [773, 1493]	0.54
Mayo Imaging Classification[2]			
1A	0 (0%)	0 (0%)	0.21
1B	2 (6%)	2 (6%)	
1C	16 (50%)	8 (25%)	
1D	8 (25%)	14 (44%)	
1E	6 (19%)	8 (25%)	
Blood Pressure (mmHg)[2]			
Systolic	126 ± 13	126 ± 10	0.96
Diastolic	77 ± 8	80 ± 8	0.26
Liver Function Tests			
AST[3] (U/L)	22 [19, 27]	21 [18, 23]	0.06
ALT[4] (U/L)	22 [18, 27]	19 [15, 28]	0.12
Bilirubin Total (mg/dL)	0.6 [0.5, 0.7]	0.6 [0.4, 0.8]	0.77
Serum Creatinine (mg/dL)	1.3 ± 0.5	1.2 ± 0.6	0.47
eGFR[5] (mL/min/1.73 m^2)	67 ± 27	72 ± 30	0.58
Urine Specific Gravity	1.009 [1.005, 1.012]	1.011 [1.007, 1.016]	0.11
Spot Uosm (mOsm/kg)	256 [168, 460]	482 [228, 571]	0.07
Tolvaptan Morning Dose	90 [90, 90]	90 [56, 90]	0.02
Tolvaptan Evening Dose	30 [30, 30]	30 [26, 30]	0.04

[1]Body mass index; [2]Blood pressure was not recorded in 4 subjects at tolvaptan initiation; [3]Aspartate aminotransferase; [4]Alanine transaminase; [5]Estimated glomerular filtration rate.

Supplemental Table S2. Multivariate Logistic Regression Models of Parameters Associated with Responder and Non-responder Status. Variables were selected based on a hybrid approach. The optimal model was selected based on the Akaike Information Criterion.

Model	AIC[1]	Variable	Coefficient	Stdard Error	95% Confidence Interval	*p*-value	VIF[2]
Status ~ Baseline TKV Growth Rate + Baseline Spot Uosm	**35.0**	(Intercept)	4.2	1.6	[1.1, 7.3]	0.01*	-
		Baseline TKV Growth Rate	-0.43	0.19	[-0.81, -0.06]	0.02*	1.14
		Baseline Spot Uosm	-0.006	0.004	[-0.014, -0.001]	0.03*	1.14
Status ~ Baseline Spot Uosm + Age at Tolvaptan Initiation + Gender	44.3	(Intercept)	0.3	2.0	[-3.6, 4.2]	0.88	-
		Age at Tolvaptan Initiation	0.03	0.05	[-0.06, 0.13]	0.49	1.18
		Gender Male	0.50	0.85	[-1.18, 2.17]	0.56	1.13
		Baseline Spot Uosm	-0.007	0.003	[-0.014, -0.001]	0.02*	1.25
Status ~ Baseline Spot Uosm + Age at Tolvaptan Initiation	42.6	(Intercept)	0.7	1.9	[-3.0, 4.3]	0.73	-
		Age at Tolvaptan Initiation	0.03	0.05	[-0.06, 0.12]	0.57	1.14
		Baseline Spot Uosm	-0.007	0.003	[-0.013, 0.001]	0.02*	1.14
Status ~ Baseline TKV Growth Rate + Baseline Spot Uosm + Age at Tolvaptan Initiation	37.0	(Intercept)	3.9	2.7	[-1.4. 9.1]	0.15	-
		Baseline TKV Growth Rate	-0.43	0.19	[-0.81, -0.05]	0.03*	1.16
		Age at Tolvaptan Initiation	0.01	0.06	[-0.11, 0.13]	0.89	1.44
		Baseline Spot Uosm	-0.008	0.004	[-0.016, 0.000]	0.05	1.51

[1]Akaike Information Criterion; [2]Variable Inflation Factor; * $p \leq 0.05$.
